# Multimodal neuroimaging computing: a review of the applications in neuropsychiatric disorders

**DOI:** 10.1007/s40708-015-0019-x

**Published:** 2015-08-29

**Authors:** Sidong Liu, Weidong Cai, Siqi Liu, Fan Zhang, Michael Fulham, Dagan Feng, Sonia Pujol, Ron Kikinis

**Affiliations:** 1 School of IT, The University of Sydney, Sydney, Australia; 2 Surgical Planning Laboratory, Harvard Medical School, Boston, USA; 3 Department of PET and Nuclear Medicine, Royal Prince Alfred Hospital, and the Sydney Medical School, The University of Sydney, Sydney, Australia; 4 Med-X Research Institute, Shanghai Jiao Tong University, Shanghai, China

**Keywords:** Multimodal, Neuroimaging, Neuropsychiatric

## Abstract

Multimodal neuroimaging is increasingly used in neuroscience research, as it overcomes the limitations of individual modalities. One of the most important applications of multimodal neuroimaging is the provision of vital diagnostic data for neuropsychiatric disorders. Multimodal neuroimaging computing enables the visualization and quantitative analysis of the alterations in brain structure and function, and has reshaped how neuroscience research is carried out. Research in this area is growing exponentially, and so it is an appropriate time to review the current and future development of this emerging area. Hence, in this paper, we review the recent advances in multimodal neuroimaging (MRI, PET) and electrophysiological (EEG, MEG) technologies, and their applications to the neuropsychiatric disorders. We also outline some future directions for multimodal neuroimaging where researchers will design more advanced methods and models for neuropsychiatric research.

## Introduction

Neuroimaging has advanced rapidly in the past two decades. The advanced non-invasive neuroimaging techniques, e.g., magnetic resonance imaging (MRI), positron emission tomography (PET), electroencephalography (EEG), and magnetoencephalography (MEG), have enabled the visualization and analysis of the brain function and structure in unprecedented detail and transformed the way we study the nervous system under normal and pathological conditions  [1], particularly neuropsychiatric disorders including neurological and psychiatric disorders that affect the nervous system  [2–4].

In the US, President Obama’s announcement of the ‘Brain Research through Advancing Innovative Neurotechnologies (BRAIN) Initiative’ on his state of the union address on April 2013 fueled resurgent interest in the neuroscience with a bold commitment to better understand the brain over the forthcoming decade [4]. Similar projects have been undertaken in the European Union [5] and Asia  [6].

Multimodal neuroimaging, which we declare as the summation of information from different neuroimaging modalities, has become one of the major drivers in neuroimaging research due to the recognition of the clinical benefits of multimodal data [7, 8], and the better access to hybrid devices, e.g., PET/CT   [9, 10], PET/MRI  [11], and PET/MRI/EEG [12]. Multimodal neuroimaging data can either be obtained from simultaneous imaging measurement (EEG/fMRI [13], PET/CT[14]), or integration of separate measurements (PET and sMRI [15], sMRI and dMRI [16], fMRI and dMRI [17]).

Multimodal neuroimaging advances neuroscience research, i.e., neurology, psychiatry, neurophysiology, and neurosurgery, by overcoming the limitation of individual modalities and by allowing a more comprehensive picture of the brain. For instance, we can jointly analyze the structure and function using the data provided by PET/CT and PET/MRI; EEG combined with functional MRI (fMRI) improves the spatiotemporal resolution that cannot be achieved by the single modality alone. Multimodal neuroimaging can also cross-validate findings from different sources and identify associations and patterns, e.g., causality of brain activity can be deduced by linking dynamics in different imaging readings. It can provide access, in an experimental setting, to determine the roles of different brain areas from multiple perspectives.

The growth of neuroimaging has spurred a parallel development of multimodal neuroimaging computing, which focuses on computational analysis of multimodal neuroimaging data, including pre-processing, feature extraction, image fusion, machine learning, visualization, and post-processing. These computational advances help to address the variations in spatiotemporal resolution and merge the biophysical/biochemical information in images  [18].

**Fig. 1:**
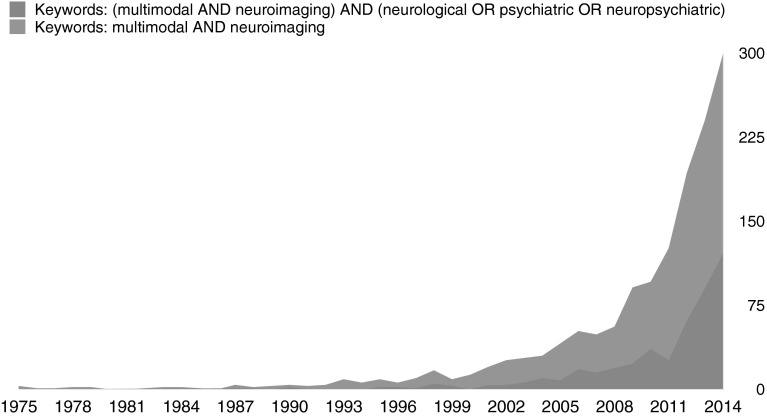
The explosive growth of multimodal neuroimaging studies over the past two decades. (Color figure online)

We conducted a search on PubMed using the keywords ‘multimodal AND neuroimaging’ up to ‘31 Dec 2014.’ There were 1461 relevant publications retrieved from the database. Figure 1 illustrates how multimodal neuroimaging in neuroscience research has rapidly expanded over the past 10 years. In 2004, there were 30 publications, and in 2014, there were close to 300 (indicated by the green area). There is a wide range of applications of multimodal neuroimaging, clinical and non-clinical, including building a brain machine interface (BMI)  [19], tracing neural activities and information pathways  [20], mapping mind and behavior to brain regions [21–23], evaluating the effects of pharmacological treatments  [24, 25], and image-guided therapy (IGT)  [26–28].

An important clinical application is the provision of functional and anatomical data for diagnosis of neuropsychiatric disorders  [3, 4]. In another PubMed search on these 1461 publications, using the keywords ‘(multimodal AND neuroimaging) AND (neuropsychiatric OR neurological OR psychiatric),’ a substantial proportion (over 30%) of the relevant results focused on the neuropsychiatric disorders (see blue area in Fig. 1). The number of publications dramatically increased each year from 10 to 121 in the period 2004–2014.

Previous reviews mainly focused on a single neuropsychiatric disorder, and summarize the image-based findings of them. For Alzheimer’s disease (AD), for example, Perrin briefly reviewed the multimodal techniques, including PET, fMRI, structural MRI (sMRI), and biochemical examination of cerebrospinal fluid (CSF), to detect AD pathology  [29]. Ewers et al. integrated the findings on changes in cortical gray matter volume, white matter fiber tracts, and brain metabolism of patients  [30], and discussed the sequential changes in neuroimaging biomarkers during different disease stages  [31], similar to the review of Lin et al. [32]. In a more recent review, Nasrallah et al. extended a review to other forms of neurodegenerative dementia  [33]. More in-depth reviews on other neuropsychiatric disorders can be found in Sect. 3.

The goal of this review differs from those above in that our interest is to review the recent advances in multimodal neuroimaging and evaluate its applications in neuropsychiatric disorders. Such a review will provide a clearer picture of the current status and offer insights and inspiration to researchers as they design better models/methods for future research.

An extensive review of the image-based findings in neuropsychiatric disorders is beyond the scope of this paper, and we instead review recent studies with a focus on the applications of multimodal neuroimaging, and refer the readers to other reviews for the detailed findings. In Sect. 2, we provide an overview of the common multimodal neuroimaging techniques, and analyze the spatial/temporal resolution, functional/structural connectivity, sensitivity/specificity to brain changes, risks/benefits for clinical applications, computing workflows, and future potential. In Sect. 3, we discuss how these neuroimaging techniques can complement each other, and how they are applied in neuropsychiatric disorders. In Sect. 4, we outline future directions for multimodal neuroimaging in neuropsychiatric research.

## An overview of neuroimaging techniques

The different neuroimaging techniques have different biophysical/biochemical mechanisms, and vary in imaging capabilities. Current neuroimaging techniques could be broadly classified into functional and structural neuroimaging. For example, sMRI reveals the detailed anatomy of the brain, and diffusion MRI (dMRI) provides information about fiber tracts. Functional modalities, including fMRI, PET, and EEG/MEG, provide data in brain metabolism and neural activity.

In the following paragraphs, we briefly summarize these neuroimaging techniques with respect to

- spatial resolution; exploring the brain anatomy and detecting morphological changes
- temporal resolution; monitoring neural activities and interactions, tracing information pathways
- structural connectivity; tracing the major brain white matter pathways
- functional connectivity; recording the neural co-activation, in the resting state
- molecular imaging; detecting the molecular activity using agents to target specific functions
- safety and risks
- clinical availability, accessibility, and ease of use
- future developments

**Fig. 2:**
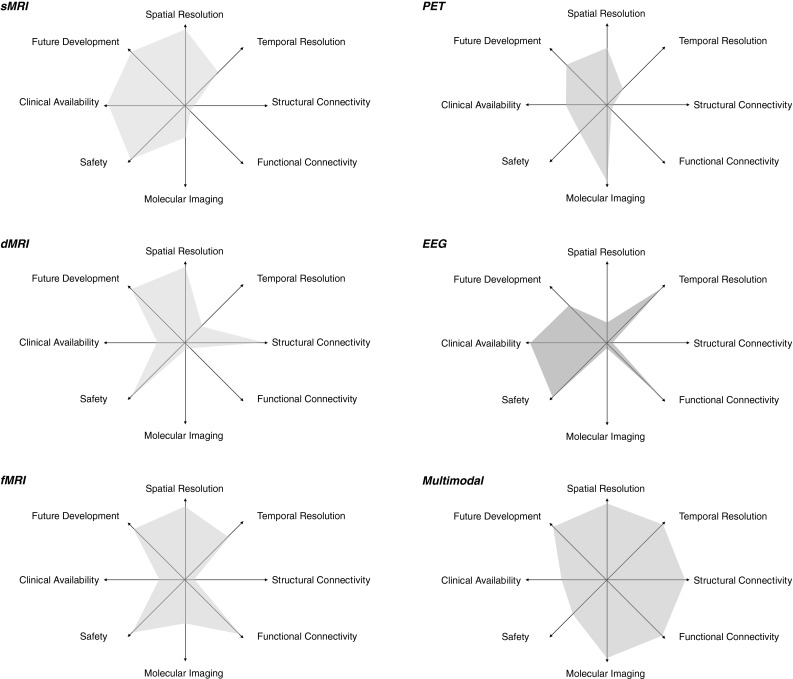
The overview of the properties of sMRI (*blue*), dMRI (*green*), fMRI (*orange*), PET (*red*), EEG (*violet*), and multimodal neuroimaging (*gray*), as indicated by the polar diagrams. *Each axis in the diagram* represents an attribute, and greater distance from the origin means better performance. Note the indexes in the diagrams are merely indicative and should not be interpreted in a quantitative way. (Color figure online)

### Structural MRI (sMRI)

sMRI includes a range of sequences—T1, T2, FLAIR, proton density [34]—that provide detailed information of brain structure, and sMRI is critical for the management of neuropsychiatric disorders. sMRI has spatial resolution up to 0.32 mm (isotropic)  [12]. As shown in Fig. 2, sMRI, however, does not provide connectivity information. Currently, there are approximately 25,000 MRI scanners in use worldwide  [35]. MRI is generally a safe procedure in patients who do not have implanted devices, such as pacemakers and implantable defibrillators  [36], although there are new MRI compatible pacemakers/defibrillators that have been introduced  [37]. MRI uses magnetic and radio waves to generate images, rather than ionizing radiation like X-ray or gamma ray. There are no known harmful side-effects associated with temporary exposure to strong magnetic field and radio waves used by MRI scanners. The narrow bore of MRI scanners is problematic for patients who are claustrophobic or overweight.

When certain contrast agents, mainly iron-oxide-based, are used, sMRI can detect the activity of the targeted molecules with high sensitivity and specificity. Gauberti et al. recently gave a detailed review of the recent advances in ‘molecular’ MRI highlighting molecules that play an important role in neuroinflammation and which may be used as therapeutic targets and biomarkers for neurological disorders [38]. sMRI is a mature technique used in scientific and clinical applications for decades; yet there are still many new developments, i.e., new pulse sequences, new contrast agents, ultra-high magnetic field, and hybrid scanners, all of which offer new imaging opportunities.

### Diffusion MRI (dMRI)

dMRI is a MRI sequence that encodes molecular diffusion effects in the nuclear magnetic resonance signal by using bipolar magnetic field gradient pulses [39]. DTI is a form of diffusion imaging where fiber tracts can be delineated based on the fractional anisotropy  [40] and is currently the only technique that allows us to trace the brain white matter pathways in vivo, as shown in Fig. 2—dMRI. By probing at many different orientations, dMRI is able to estimate the orientation of axonal fiber bundles, based on the fact that water diffuses most rapidly along the length of axons. This also leads to longer scanning time as compared to sMRI.

Currently, dMRI is used as a research tool in laboratories, and has not been evaluated in clinical trials due to the crossing-fiber problems, the differences in signal estimation models and fiber tracking algorithms, the variations in datasets, and the lack of ground truth. Nevertheless, DTI is used clinically in the pre-operative planning prior to surgical resection of gliomas which usually displace but can involve the fiber tracts. New models and methods are proposed each year, e.g., the q-space trajectory imaging (QTI)  [41]. Large-scale datasets with uniformly collected dMRI data are also growing in size, and will facilitate the evaluation of these models and methods  [42].

### Functional MRI (fMRI)

fMRI is a MRI technique that can depict brain activity by detecting the associated changes in brain hemodynamics. It uses blood-oxygen-level-dependent (BOLD) contrast that is closely related to cerebral blood flow (CBF), as brain function requires blood flow to supply oxygen for energy consumption by neurons. It has relatively high spatial resolution (2mm isotropic) and medium temporal resolution (minutes) for a set of successive scans. Similar to sMRI, fMRI can be used to label specific molecules with contrast agents  [43], i.e., molecular fMRI  [44]. fMRI is used clinically to identify eloquent cortex prior to surgery, e.g., identifying the motor cortex prior to resection of a glioma in the posterior frontal lobe. Two particular strengths of fMRI are that it is able to detect brain activation induced by a task, and provide the connectivity between populations of neurons based on their co-activation at resting state. These two benefits essentially define the two categories of fMRI analyses, task-evoked fMRI and resting-state fMRI.

When the brain is performing a task, CBF usually changes as neurons work to complete the task. The primary use of task-evoked fMRI is to identify the correlation between brain activation/interaction pattern and cognitive states, such as perception, language, memory, emotion, and thought [45, 46]. Recent research based on task-evoked fMRI indicated that altered cognitive functions are related to neuropsychiatric disorders. For instance, emotion regulation capability is not sustained in depressed patients as compared to healthy control subjects [47]. Resting-state fMRI is used to detect the spontaneous activation pattern in the absence of an explicit task or stimuli [48]. Resting-state fMRI enables us to deduce the functional connectivity between dispersed brain regions, which form functional brain networks, or resting-state networks (RSNs). The default mode network (DMN) is a functional network of several brain regions that show increased activity at rest and decreased activity when performing a task  [49]. DMN has been widely used as a measure to compare individual differences in behaviors, genetics, and neuropathologies, although the use of it as a biomarker is controversial  [50, 51].

Recent improvements in spatiotemporal resolution of fMRI have led to higher statistical power to detect RSNs. Further investigation is needed to derive the neuropsychiatric biomarkers from the network and/or network dynamics, and further evaluate them for the diagnosis of individual neuropsychiatric disorders and to guide therapy.

### Positron emission tomography (PET)

PET is the most powerful and versatile approach to study neurotransmitter/receptor interactions. It has lower spatiotemporal resolution when compared to MRI, and involves injection of a radioactive tracer and exposure to ionizing radiation. PET is inherently a molecular imaging technique, which is exquisitely sensitive for detecting the targeted molecules or processes. For example, *2-*[$^{18}$F]*fluoro-2-deoxy-D-glucose* (FDG) is the most widely used radiotracer that can assess the glucose metabolism in brain, thus has been used for diagnosis, staging, and monitoring treatment of cancers  [52] and neurodegenerative disorders  [53]. In recent years, the percentage of FDG-PET brain studies have decreased due to the introduction of new tracers, e.g., the amyloid-binding compounds, $^{18}$F-BAY94-9172, $^{11}$C-SB-13, $^{11}$C-BF-227, $^{18}$F-AV-45, and $^{11}$*C-Pittsburgh compound B* ($^{11}$C-PiB). There are a number of reviews of amyloid imaging agents  [29, 54–56].

### Electroencephalography (EEG) and magnetoencephalography (MEG)

EEG and MEG detect the synchronized activity of an assembly of neurons by displaying their weighted sum of instantaneous neuronal electrical current or magnetic fluxes throughout the brain. EEG and MEG are widely used in neurology clinics due to the simplicity and mobility of EEG monitoring systems, both are safe procedures. EEG and MEG allow us to explore brain cortical activation pattern with ultra-high temporal resolution and record the event-evoked neural information flow in real time. However, EEG and MEG are limited by the low spatial resolution and specificity, and the inability to detect and record the signals from subcortical regions.

An important opportunity for the future is the integration of EEG and MEG with MRI, in particular, with fMRI. EEG and MEG are able to demonstrate the brain activation at much greater temporal resolution when compared to MRI. The MRI produces the anatomical template which enhances the inherent poor spatial resolution of EEG and MEG source images. However, a major challenge has been to develop EEG and MEG technology that can operate in a high magnetic field. Other challenges exist to better understand the correlation between BOLD signals and electrophysiological events via neurovascular coupling and enhance performance of EEG source imaging from simultaneously acquired fMRI data.

### Multimodal neuroimaging

Multimodal neuroimaging, which we refer to as the collective information offered in multiple imaging modalities, has become a major driver for current research due to the awareness of the clinical benefits of the multimodal data. As shown in Fig. 2, multimodal data analysis could take the advantages from multiple imaging techniques, e.g., improving both spatial and temporal resolution, finding the anatomical basis for functional connectivity, targeting disease biomarkers with high specificity and sensitivity, along with many new opportunities to improve brain research. Multimodal neuroimaging is currently limited by the availability and safety of the imaging scanners, but novel neuroimaging scanners, especially the hybrid scanners, such as PET/CT and EEG/MRI, will become more widely available in the midrange future. Multimodal neuroimaging analysis is much more challenging than single modality analysis, as multimodal neuroimaging requires sophisticated computing methods, i.e., pre-processing, feature extraction, image fusion, machine learning, visualization, and post-processing, to tackle the large variations in the spatiotemporal resolution and integrate the biophysical/biochemical information of the multimodal data. Many multimodal neuroimaging computing methods have been proposed and applied to a wide range of clinical and non-clinical applications, e.g., brain computer communication  [19], information pathways tracing  [20], brain mapping  [21–23], drug development and discovery  [24, 25], pre-operative surgical planning, and intra-operative surgical navigation  [28, 57].

As shown in Fig. 1, there has been explosive growth in multimodal neuroimaging approaches during the last decade, and we may foresee such growth in the following years.

## Applications to neuropsychiatric disorders

**Fig. 3:**
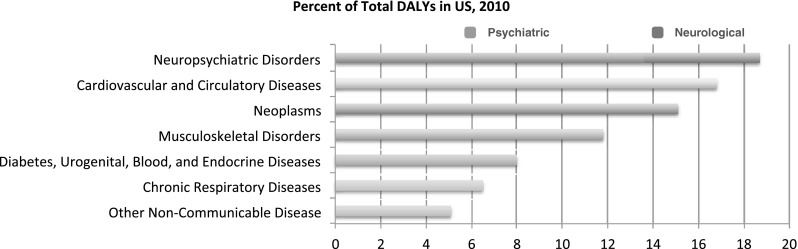
The disability-adjusted life years (DALYs) of 291 diseases and injuries based on the systematic analysis of descriptive epidemiology from 1990 to 2010 in US [58]. (Color figure online)

Neuropsychiatric disorders represent the most disabling and costly category, based on the systematic analysis of descriptive epidemiology of 291 diseases and injuries from 1990 to 2010 for 187 countries  [58]. As shown in Fig. 3, neuropsychiatric disorders caused the largest number of years lost due to illness, disability, and early death measured by disability-adjusted life years (DALYs) in US, and the socioeconomic burden of neuropsychiatric disorders will be aggravated as people live longer.

**Fig. 4:**
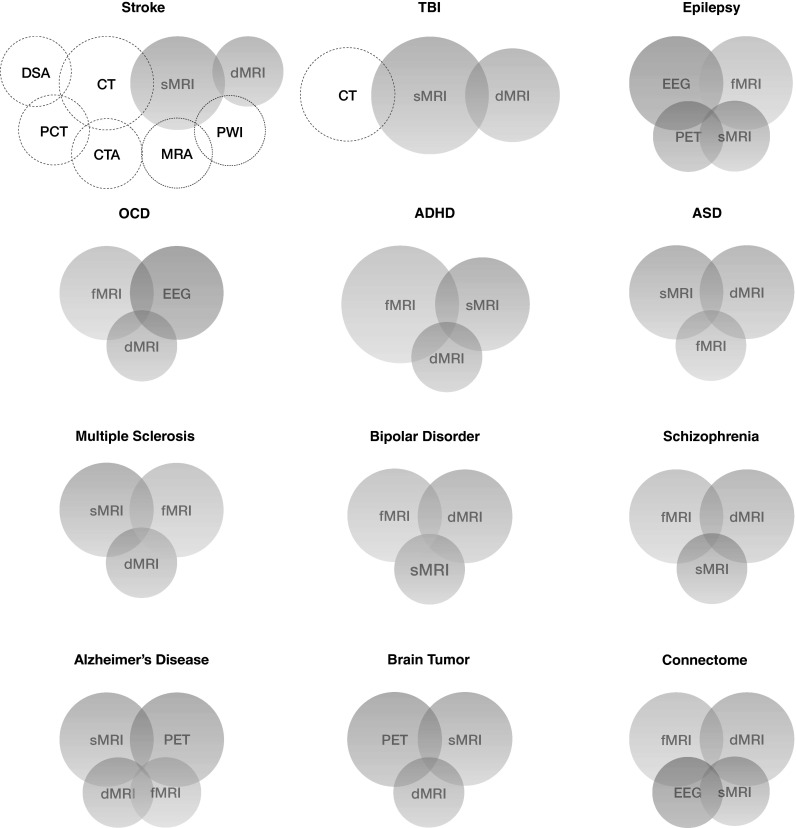
The applications of the multimodal neuroimaging approaches in a variety of neuropsychiatric disorders, as well as in stroke, brain injury, brain tumor, and connectome. The *color of circle* indicates various neuroimaging techniques, same as in Fig. 2. The *size of the circle* indicates the prevalence of use the technique in specific applications. Note the sizes are only indicative and should not be interpreted in a quantitative way. (Color figure online)

Neuroimaging techniques have expanded beyond a traditional diagnostic role to have a fundamental role in patient management from diagnosis, to selection and assessment of treatment and to prognosis stratification. There is a rising trend of using the multimodal neuroimaging approaches in neuropsychiatric disorders, as shown in Fig. 1. In this section, we summarize how these neuroimaging techniques can be integrated using the multimodal computing methods, and further demonstrate their applications in neuropsychiatric disorders as well as in stroke, traumatic brain injury (TBI), brain tumors, and the brain connectome (Fig. 4).

These multimodal approaches can be separated into categories that include a structural–structural combination, a functional–functional combination, and a structural–functional combination. Each category has different applications, and requires different computing workflows. In brief, a structural–structural combination, e.g., sMRI-dMRI, is used to extract and fuse various morphological features and is applied to disorders that affect both gray matter and white matter, such as TBI and stroke. The functional–functional combination can be used to explore brain activation/metabolism patterns and is mainly applied to cognition and consciousness-related disorders, e.g., epilepsy and obsessive-compulsive disorder (OCD). The structural–functional combination is virtually applicable to all disorders, but more frequently used for identifying the structure–function associations in neurodegenerative disorders, neurodevelopmental disorders, multiple sclerosis, schizophrenia, bipolar disorder, brain tumors, and the brain connectome.

### Structural–structural combination

*sMRI-dMRI* methods dominate the structural–structural category, as they take clinical benefits of sMRI and dMRI by integrating the gray matter and white matter morphometry. It has become a useful tool to detect lesions and evaluate treatments for various neuropsychiatric disorders that cause brain morphological changes. Here, we list a few examples of clinical uses of sMRI-dMRI.

Traumatic brain injury (TBI) has very high incidence, resulting in 6.8 million TBI cases every year in the US, and causes impairment of memory, information processing, attention, and executive function  [59]. Multimodal structural neuroimaging can assist neurosurgeons, intensive care specialists, neurologists, and rehabilitation specialists in the management of TBI  [60]. Conventional brain CT usually fails to detect the subtle structural abnormalities in mild TBI, and sMRI and dMRI are the methods of choice to evaluate and predict outcome in TBI. The sMRI sequences (T1, T2, FLAIR, susceptibility-weighted imaging (SWI) and gradient-recalled echo (GRE)) provide highly accurate depiction of pathological lesions, and dMRI detects the effects of TBI on brain connectivity and non-hemorrhagic diffuse axonal injury (DAI), which are not detected by CT. The sMRI-dMRI methods are widely used in TBI  [61, 62]. There are also some studies that have used dMRI and fMRI to validate the connectivity information in TBI patients in the recovery phase  [63, 64].

The sMRI-dMRI methods have been routinely used in the assessment and treatment planning for stroke. Stroke is a leading cause of death worldwide. There are different types of stroke, and each requires a different diagnostic approach and treatment. T2*-weighted sMRI, e.g., SWI and GRE, is primarily used to detect hemorrhagic stroke, and has equal sensitivity to standard CT methods. However, dMRI is 4-5 times more sensitive in detecting acute ischemic stroke than CT. Other structural imaging techniques, such as perfusion CT (PCT), CT angiography (CTA), digital subtraction angiography (DSA), perfusion-weighted imaging (PWI), and MR angiography (MRA), can also be used to evaluate suspected vascular occlusion, edema, and cerebral infarction. Tong et al. [65] recently published a comprehensive comparison of these methods in the evaluation and management of stroke. Another review on multimodal neuroimaging in stroke is given by Copen et al.  [66].

sMRI-dMRI methods have also been used to analyze the gray and white matter alterations in schizophrenia  [67] and Autism spectrum disorders (ASDs)  [16, 68], neurodegeneration simulation  [69], classification of AD and frontotemporal dementia (FTD)  [70], and Parkinson’s Disease (PD) staging  [71].

### Functional–functional combination

*EEG-fMRI* is valued in functional brain research due to the complementary nature of EEG and fMRI. EEG-fMRI can provide simultaneous cortical and subcortical recording of brain activity with high spatiotemporal resolution.

Epilepsy is one of the most prevalent neurological disorders worldwide. EEG-fMRI is increasingly used to provide clinical support for the diagnosis of epilepsy, in addition to the routinely used sMRI  [72] and PET  [14, 73]. Researches have used EEG-fMRI to identify a set of brain functional regions that collectively form ‘consciousness,’ including contributions from the DMN, ascending arousal systems, and the thalamus, as summarized by Bagshaw et al.  [74]. The activation of these regions and the connection of the networks are important in the evaluation of epilepsy, and together may provide a more fundamental understanding of the alterations of consciousness experienced in epilepsy. Abela et al.  [75] focused on altered network compositions in epilepsy, and identified the specific connectivity pathways that characterize the underlying epilepsy syndromes, such as mesial temporal lobe epilepsy (MTLE), lateral temporal lobe epilepsy (LTLE), frontal lobe epilepsy (FLE), idiopathic generalized epilepsy (IGE), and absence epilepsy (AE). A substantial proportion of patients have refractory epilepsy and surgery offers the potential to reduce seizure frequency. Successful surgical treatments, however, require accurate localization of the seizure onset zones and an understanding of surrounding functional cortex to avoid iatrogenic disability. PET, MRI, and intracranial EEG (iEEG) are all needed for optimal surgical planning and treatment evaluation of refractory epilepsy  [76, 77].

Another important application of EEG-fMRI is to evaluate patients with obsessive-compulsive disorder (OCD). OCD is a chronic and relatively common neuropsychiatric disorder that characterized by stereotyped and repetitive behaviors. Patients with OCD feel intense need to carry out these behaviors, and have impaired ability to recognize an error and to adjust future responses. OCD may result in social disability. Two neuroimaging biomarkers of error commission, the error-related negativity (ERN) and the dorsal anterior cingulate cortex activation, have been identified using EEG and fMRI, respectively  [78]. However, Agam et al.  [79] recently suggested that these biomarkers have different neural and genetic mediation. dMRI is also increasingly being used to examine the microstructural integrity of white matter in OCD patients, since white matter abnormalities have long been suspected in OCD, but the findings are inconsistent. For example, one recent study indicated that patients with OCD had decreased fractional anisotropy in the anterior cingulum bundle  [80], but in another recent study, the OCD patients showed increased fractional anisotropy of the cingulum bundle  [81]. Further investigation on large datasets is needed to confirm these findings.

### Structural–functional combination

*sMRI-dMRI-fMRI* has been ubiquitously used in neuropsychiatric research largely because of high clinical availability, and partially due to its capability to link brain function, structure, and connectivity. It has been increasingly used in research in attention-deficit hyperactivity disorder (ADHD), Autism spectrum disorder (ASD), bipolar disorder, schizophrenia, and clinically in multiple Sclerosis (MS).

ADHD is one of the most commonly diagnosed childhood behavioral disorders. It is characterized by persistent inattention (ADHD-I), hyperactivity-impulsivity (ADHD-H), or a combination of both (ADHD-C). ADHD affects at least 5–11% of school-age children, and symptoms may persist into adulthood  [82]. Previous studies using sMRI have reported various findings, such as decreased total brain volume and abnormalities in specific brain regions. The task-evoked and resting-state fMRI approaches were also used in ADHD studies to detect the abnormal brain activation. The use of sMRI and fMRI was reported recently in ADHD  [83, 84]. It is only quite recently that dMRI has been applied to ADHD to characterize the disrupted interconnected structural networks in the brain. Shenton et al. provided a brief summary of the latest studies  [85]. For example, Hong et al. used dMRI and whole-brain tractography to investigate the altered white matter connectivity in 71 children with ADHD, and identified a single network (comprising 23 brain regions and 25 links) that differentiates the ADHD group from the normal control group  [86].

ASDs are neurodevelopmental disorders characterized by deficits in social reciprocity, impaired communication, and restricted interests and repetitive behaviors. Previous studies using sMRI have shown that infants with ASD might have excessive brain growth followed by abnormally slow or even arrested growth as compared to normal developing control infants in early childhood  [87]. Subsequent research indicated ASD affects both gray and white matter volumes. Therefore, dMRI has been exploited to describe the microstructural integrity and orientation of white matter. fMRI has enhanced the understanding of the neural circuity of ASDs by demonstrating the convergent structural and functional changes  [88, 89]. For example, Mueller et al. used sMRI-dMRI-fMRI approach and identified three brain areas with strong correlations between the structural and functional abnormalities: right temporoparietal junction and the left frontal lobe, bilateral superior temporal gyri, and the right temporoparietal region  [90].

MS is a demyelinating disease commonly seen in young people. The cause of MS is unknown. Symptoms and signs vary across patients and can include cognitive impairment, fatigue, vertigo, diplopia, ataxia, hemiparesis, and paraparesis in severe MS patients. Histopathologic and neuroimaging examinations suggest that both white matter and gray matter are affected. In particular, the thalamus can be affected frequently in MS  [91], which can lead to impaired cognition. sMRI can detect the thalamic atrophy; dMRI can be used to demonstrate the altered thalamocortical white matter pathways, and fMRI can be used to show the association between the resting-state thalamocortical functional connectivity and cognitive impairment. Recently, sMRI-dMRI-fMRI was jointly used in several studies  [92, 93].

Bipolar disorder is a psychotic disorder that characterized states of depression and mania, and sometimes with symptoms common to schizophrenia. It is therefore difficult to conceptualize bipolar disorder and its subtypes, and differentiate it from other psychiatric disorders. The multimodal MRI methods have been applied to bipolar disorder and clearly demonstrate abnormalities in brain networks associated with emotion processing, emotion regulation, and reward processing. In a recent study, Sui et al. proposed a joint analysis model for fMRI and DTI for discriminating bipolar disorder from schizophrenia  [94]. Common abnormalities were seen in dorsolateral prefrontal cortex, thalamus, and uncinate fasciculus, whereas differences were found in medial frontal and visual cortex, as well as occipitofrontal white matter tracts. Phillips and Swartz recently published an extensive review of these neuroimaging findings and further pointed out the future directions of neuroimaging research in bipolar disorder  [95].

Schizophrenia is a major psychosis that is characterized by altered perception, thought processes, and behaviors. It can be highly heritable disorder  [96], and can be triggered by a combination of genetic factors and environmental interactions  [97]. Disconnection in white matter pathways and alteration of cortex are assumed to underlie the cognitive abnormalities in schizophrenia, although this is a hypothesis and as yet there is no direct proof. The approaches used for characterizing schizophrenia are very similar to those for bipolar disorder, primarily using sMRI-dMRI-fMRI. Various findings in schizophrenia studies have been reported, based on the investigation on microstructure of white matter  [98] or gray matter  [97], or the connectivity between different brain regions  [67, 99].

The study of brain networks, the connectome, is the focus of intense current neuroscience research [100]. Exploration on the neural systems and brain connections is critical to advance our understanding of normal brain reaction and is one of the greatest challenges of the twenty first century. The Human Connectome Project^1^ is directed at tackling this challenge using the highest quality imaging data available today, predominantly MRI data, complemented by EEG and MEG. The information about brain anatomy, structural connectivity, and functional connectivity is being obtained using dMRI and resting-state fMRI. Additional information about brain function is being obtained using task-evoked fMRI, EEG, and MEG to record the brain activity.

*sMRI-PET* is a new structural–functional combination that is being applied to neurodegenerative diseases and brain tumors to improve the localization and targeting of diseased tissue with high accuracy and sensitivity. AD is the most common neurodegenerative disorder among aging people, and it accounts for close to 70% of all dementia cases. In AD, activities of daily living deteriorate over a number of years, ultimately leading to death. There is no cure [101]. AD neuroimaging biomarkers can detect the changes in brain structure (e.g., atrophy on sMRI) and function (e.g., hypometabolism, amyloid plaque, and NFT formation on PET) before there is cognitive impairment. As a result, sMRI and PET with $^{18}$F-FDG and amyloid tracers are being increasingly used in the evaluation of patients with early dementia in the research setting  [8, 102–106]. These studies also demonstrated clear benefits of multimodal neuroimaging over any single technique alone. Recently, dMRI  [107, 108] and fMRI  [109] have also been used in the evaluation of dementia as there is evidence that suggests the functional connection between networks is disrupted  [110–112]. There are many extensive reviews which summarized these imaging techniques and the image-based findings [29–31, 33].

Over 200,000 individuals are diagnosed with primary or metastatic brain tumors in the US each year  [28]. The primary use of sMRI-PET in brain tumors is to accurately localize and label the lesion, e.g., tumor and edema. PET has the potential to more accurately detect the peripheral tumor boundary than using sMRI alone  [11, 113]. For brain tumor surgery, dMRI is usually combined with sMRI and PET for pre-operative surgical planning and intra-operative surgical navigation. For example, Durst et al. used dMRI to predict tumor infiltration in patients with gliomas  [114]. Tempany et al. used sMRI and dMRI tractography to display a complete brain map for surgical planning  [28]. They further demonstrated how to optimize the separation between tumor and normal brain in intrinsic brain tumors with sMRI, and how to avoid inadequate resection of the tumor.

## Future directions

Multimodal neuroimaging approaches have been increasingly used in detection, diagnosis, prognosis, and treatment planning of neuropsychiatric disorders. In this paper, we have briefly summarized the recent advances in neuroimaging techniques, and reviewed their applications to neuropsychiatric disorders to provide an overview of the current status. We have also outlined some future directions for multimodal neuroimaging research.

*Improved neuroimaging capabilities* Neuroimaging techniques will continue to advance rapidly, with higher spatial/temporal/angular resolutions, shorter scan time, and better image contrast. In particular, hybrid scanners, e.g., PET/CT and PET/MRI, will become more clinically accessible. These technologies will enable more discoveries in the neuropsychiatric disorders. The improved imaging capabilities will offer better neuroimaging biomarkers to evaluate neuropsychiatric disorders, and various subtypes or different stages of the same disorder with higher statistical power. These biomarkers will be standardized so they can be widely used clinically and evaluated in large-scale sample sets. In addition, once the biomarkers reach a satisfactory level or the treatment, appropriate clinical guidelines must be developed to support and encourage widespread clinical testing.

*Enhanced neuroimaging computing models and methods* The continued growth in the complexity and dimensionality of the neuroimaging data will spur the parallel advances of computation models and methods to analyze such complex data. Future neuroimaging analysis models will integrate the longitudinal information to track the long-term changes in the biomarkers [115]. This is essential for us to understand the pathology of the disorders and its degeneration trajectory. With sufficiently large longitudinal datasets, we may be able to identify the causes and detect the early signs, as well as predict the course of the disorders. Future studies will also focus on subject-centered therapy. However, no matter how large the datasets are, they cannot include the entire population, and there will always be inter-subject variations. Personalized/patient-centered care is highly demanded and is the ultimate goal of neuroimaging studies [116]. Neuroimaging computing models and methods also need to keep increasing the degree of automation, accuracy, reproducibility, and robustness, and eventually need to be integrated into the clinical workflow to facilitate clinical testing of the new neuroimaging biomarkers.

*Converged neurotechnologies* Another future direction will be to combine imaging with non-imaging studies. The multidisciplinary nature of neuroimaging computing will keep bringing together clinicians, biologists, computer scientists, engineers, physicists, and other researchers. Imaging genetics is a very promising area for the future, where the aim is to identify the genetic basis of anatomical and functional abnormalities of the human brain and show how this is connected with neuropsychiatric disorders. There is a trend to use imaging findings in brain disorders to reveal the endophenotypes for various gene mutations. By converting the endophenotype data to novel genetic biomarkers, it may be possible to identify individuals at greater risk of developing brain disorders, and in the near future provide treatment options before the symptoms appear.

## Footnotes

1 http://www.neuroscienceblueprint.nih.gov/connectome
